# Changing patterns in joint replacement surgery in the hand in Sweden: a population-based study of 5382 patients

**DOI:** 10.1177/17531934251331360

**Published:** 2025-04-12

**Authors:** Viktor Schmidt, Elsa Pihl, Cecilia Mellstrand Navarro, Michael Axenhus

**Affiliations:** 1 Danderyd Hand and Wrist Initiative, Danderyd Hospital, Stockholm, Sweden; 2 Department of Orthopaedic Surgery, Danderyd Hospital, Stockholm, Sweden; 3 Department of Clinical Sciences at Danderyd Hospital, Karolinska Institutet, Stockholm, Sweden; 4 Department of Clinical Science and Education Södersjukhuset, Karolinska Institutet, Stockholm, Sweden

**Keywords:** Demographic disparities, hand prosthesis, incidence rates, population-based study, predictive analysis, surgical trends, Sweden

## Abstract

Over a 16-year period (2008–2023), data from the Swedish National Patient Register reveal shifting trends in operation for hand joint replacements linked to demographic, healthcare and surgical advances. Among 5382 identified cases, 63% were women, with the incidence peaking in the 65–74 age bracket, highlighting a marked gender gap in middle and older age groups. Regional analyses indicate significant disparities, as Örebro and Halland had rates exceeding 10 per 100,000, while Stockholm and Blekinge fell below 5 per 100,000. The adoption of total prostheses without cement declined by 22%, reflecting changing surgical preferences. Predictive modelling anticipates an overall decline in incidence by 2035, with gender-specific rates converging over time. These findings highlight the need for targeted healthcare policies that address inequities and minimize unwarranted variations in treatment. Standardized care programmes that support evidence-based surgical decision-making could reduce the incidence of both over- and under-treatment.

**Level of evidence:** III

## Introduction

Joint replacement surgery is an intervention for reducing pain, restoring functionality and improving quality of life in patients with degenerative or post-traumatic hand joint conditions. Over the past several decades, advancements in surgical techniques and prosthetic technologies have transformed the field; however, comparative studies investigating the effectiveness, complications and longevity of implants are largely lacking (Athlani et al., 2024; Monir et al., 2025; Smith et al., 2024).

Previous studies on hand joint replacement surgery have primarily focused on technical advancements and postoperative outcomes, often neglecting broader epidemiological trends. Healthcare resource allocation and population demographics vary significantly across regions and national level data on this type of hand surgery is lacking.

Using large scale data from the Swedish national patient register, the aims of this study were: to analyse demographic variations in incidence rates, with a focus on age and sex differences; evaluate regional disparities to identify high- and low-incidence areas; assess temporal changes in surgical techniques and preferences; and predict future trends to inform healthcare planning.

## Methods

### Study design and data framework

This observational study used population-based data extracted from the Swedish National Patient Register (NPR), for a period from 2008 to 2023. The study adhered to the principles outlined in the RECORD guidelines (Benchimol et al., 2015).

### Healthcare infrastructure in Sweden

Sweden’s healthcare system offers universal access to residents. Each Swedish resident is assigned a unique personal identification number, which is pivotal for tracking healthcare interactions across all medical facilities (Ludvigsson et al., 2009).

### Data collection and registry details

Reporting to the NPR is mandatory for all public and private healthcare providers and includes diagnoses based on the ICD-10 coding system and surgical procedures classified under the NOMESCO framework (Nordic Medico-Statistical Committee, 2010; Steindel, 2010). The NPR has been validated confirming high accuracy of data (Klassifikationen ICD-10, 2025; Ludvigsson et al., 2011; Südow et al., 2023).

### Selection of study participants

The study focused on individuals aged 15 years and older who underwent a primary hand joint prosthesis, coded as NDB40-46 in the NOMESCO classification. Eligibility was confined to those with valid Swedish personal identification numbers.

### Statistical approach

Data were stratified by sex, surgical method, age group and geographic region to discern trends over time. Incidence rates were standardized by dividing the number of operations by population statistics obtained from Statistics Sweden (n.d.). Differences in incidence rates between sexes within each age group were analysed using Student’s *t*-tests. Regression models were fitted to the data, including exponential, linear, logarithmic and polynomial models. A best fit model was used for a predictive analysis model and results presented with 95% confidence intervals. The year was used as the independent variable and incidence per 100,000 was used as the dependent variable. Statistical significance thresholds were set at *p* < 0.05, with additional markers for varying levels of significance (*p < 0.05, **p < 0.01, ***p < 0.001, ****p < 0.0001).

### Ethical considerations

As the study used only publicly available data, no ethical approval was required per Swedish legislation.

## Results

A total of 5382 patients with a registration of prosthetic joint replacement surgery in the wrist or hand were identified. There was a consistent observation of higher procedure rates in women than men (Table 1).

**Table 1.** Total numbers for hand joint replacement operations during 2008 to 2023.

Sex	2008	2009	2010	2011	2012	2013	2014	2015	2016	2017	2018	2019	2020	2021	2022	2023
Men	101	106	111	93	99	102	94	84	93	91	65	61	51	67	70	77
Women	385	422	421	363	292	258	261	252	207	195	211	178	109	133	152	178
Men (%)	21%	20%	21%	20%	25%	28%	26%	25%	31%	32%	24%	26%	32%	34%	32%	30%

**Figure 1.**
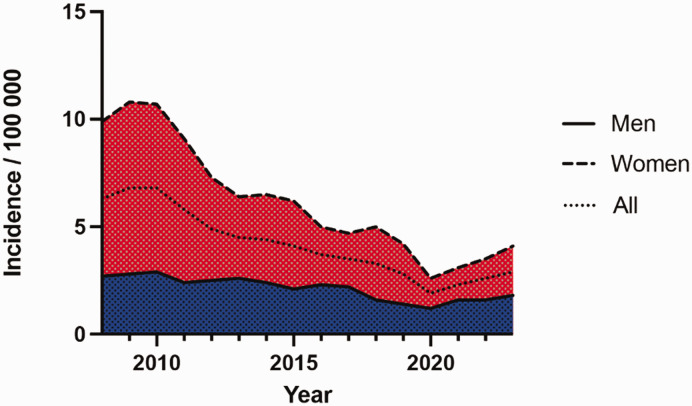
Trends in hand prosthesis surgery, 2008–2023.

The overall incidence showed a declining trend from 2010 to 2020, followed by a gradual increase in the later years. Men maintained relatively stable rates with minimal variation, whereas there were more pronounced fluctuations in women (Figure 1). The most common types of joint replacements in 2023 were trapeziometacarpal or metacarpophalangeal joints in women (Supplementary Table S1, available online).

**Figure 2.**
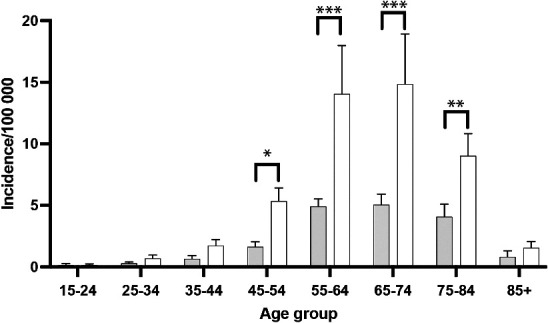
Age groups of patients who underwent primary hand prosthesis surgery during 2008–2023. White indicates women and grey indicates men (*p < 0.05, **p < 0.01, ***p < 0.001, ****p < 0.0001).

Incidence rates increased with age, peaking in the 65–74 age group for both genders, with women consistently having higher rates across all age categories. The most pronounced gender differences were observed in the 55–64, 65–74, and 75–84 age groups (Figure 2).

Regional analysis showed marked differences without restriction to urban or rural status. Regions such as Örebro, Halland and Skåne report the highest incidence rates, exceeding 10 per 100,000, while regions like Stockholm, Jämtland, and Blekinge had lower rates, under 5 per 100,000 (Supplementary Figure S1, available online).

When analysing surgical methods and choices of prostheses we noted that total prostheses without cement had the highest incidence rates, with consistently higher rates in women than men. The rates for partial implants, with or without cement, remained comparatively lower across the study period. A decline in the incidence of total prostheses without cement was observed over time for both genders, with a slight stabilization in the later years. The other categories of prosthesis, including total prostheses with cement and hybrid techniques, maintained relatively steady and low rates throughout the timeline (Supplementary Figure S2, available online).

**Figure 3.**
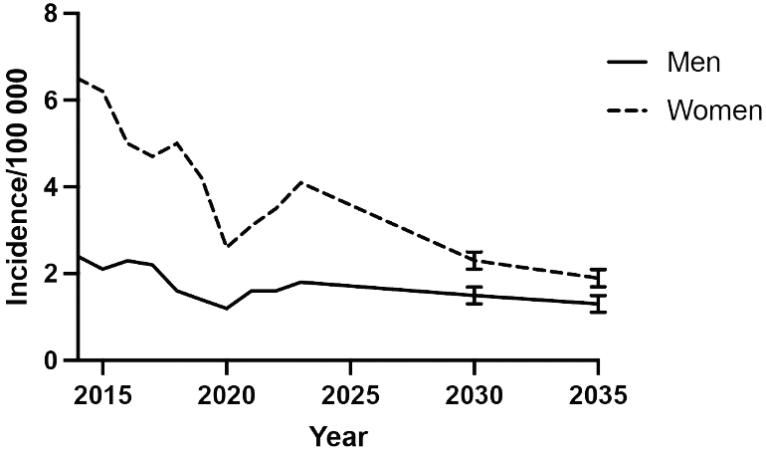
Predicted incidence rates of surgical procedures per 100,000 inhabitants for men and women to 2035. Error bars indicate 95% confidence interval.

Using predictive analysis of the retrospective data we found that the predicted incidence rates of both men and women showed a declining trend, the reduction being more pronounced in women, resulting in a gradual convergence of rates by 2035 (Figure 3).

## Discussion

This study highlights key findings on operations for prosthetic replacement hand joints in Sweden between 2008 and 2023, with significant gender and regional disparities, as well as changing trends in procedures. The higher incidence of operations in women, especially those aged 65–74, aligns with reports indicating higher rates of joint disease in women owing to rheumatic conditions, hormonal factors and longer life expectancy (Quintero et al., 2012; Tosi et al., 2024; Wolf et al., 2015). Female sex is frequently identified as a key determinant of osteoarthritis and subsequent requirement for healthcare (Harjula et al., 2018; Peshkova et al., 2022; Segal et al., 2024). Although the sex gap is expected to equalize by 2035, biological and sociocultural factors may still influence demand (Kiadaliri et al., 2019; Vogel et al., 2023).

Marked regional variations in incidence rates – over 10 per 100,000 in Örebro and Halland compared with under 5 per 100,000 in Stockholm and Blekinge – underscore uneven healthcare resource distribution, aligning with earlier Scandinavian studies (Magnéli and Axenhus, 2024; Manderbacka et al., 2022; Ponkilainenet al., 2024; Saving et al., 2018). Such disparities may reflect referral patterns, staff density, institutional practices or patient demographics, disproportionately affecting elderly populations (Fredriksson and Winblad, 2008; Jaafar et al., 2017; Kiadaliri et al., 2019; Satokangas et al., 2023a).

Comparative studies could inform targeted policies – such as expanding surgical capacity, strengthening preventive care and standardizing care programmes – to address regional inequities (Laugesen et al., 2021; Mäkelä et al., 2010). Future research should examine the socioeconomic factors, infrastructure and patient demographics that drive these variations.

The decline in cementless total joint implants reflects changing surgical practices and advancements in biomaterials. In hand surgery, there is limited evidence to compare cemented and uncemented prostheses and certain models have been withdrawn owing to inadequate long-term durability. Rigorous preclinical testing, comparative trials and mandatory monitoring are essential, highlighting the importance of prioritizing evidence-based innovation and long-term outcomes over rapid entry to the market.

There is a general trend for decreasing incidence rates; however, this probably be attributed in part to the COVID-19 pandemic and since then there has been an increasing trend. The same trend has been reported in HAKIR (the Swedish national quality register for hand surgery) (HAKIR, 2023). The predictive modelling indicates a gradual decline in overall incidence rates with a convergence in gender-specific rates by 2035. The decline until 2020 could be explained by new treatment regimens that prioritize denervation, excision procedures or arthrodesis (Chick, 2025; Jump et al., 2025). Better treatments for rheumatic diseases might also be a negative driver (Nystad et al., 2020). But future changes in medical treatment or improved surgical methods cannot be accounted for. For example the incidence of trapeziometacarpal implants seem to have been stable since 2016 but may well increase in the coming years after the introduction of newer implants that have been shown to have better longevity (HAKIR, 2023; Lussiez et al., 2021). Future research could investigate whether these trends are influenced by variations in surgical indications or patient preferences, and whether they lead to improved outcomes.

The findings of this study have direct clinical implications for surgeons, healthcare administrators and policymakers. Women in older age groups consistently show higher rates of hand joint replacement operations. The stark differences in surgical rates between regions highlight the need for equitable resource allocation. Regional disparities could be mitigated through initiatives such as mobile surgical teams or telemedicine consultations (Meara et al., 2015; Winkelmann et al., 2022). Considering the low incidence rates and the fact that procedures are technically demanding and require years of experience to master, there is a need for cautious and evidence-based adoption to avoid introducing unnecessary risks of reoperation into the healthcare system. Healthcare systems should invest in professional development programmes or nationally specialized care centres to ensure that clinicians are equipped with the skills to implement advanced prosthetic technologies effectively (Choong and Brooks, 2012; Meara et al., 2015). The projected decline in overall incidence rates, coupled with gender convergence, underscores the need for adaptable healthcare planning. Policymakers should focus on maintaining capacity for high-volume, high-quality surgical care while investing in preventative measures that address the root causes of musculoskeletal disorders, such as ergonomics programmes, early screening and public health campaigns. Interestingly, the Swedish National Guidelines for treatment of thumb base arthrosis does not recommend joint arthroplasty as a primary treatment (Nationellt vårdprogram för tumbasartros, 2022).

Sweden’s universal healthcare system ensures equitable access to surgical interventions, but disparities remain evident. This study’s findings align with research from other countries with similar systems, such as Canada and the UK, where universal access reduces financial barriers but does not eliminate regional and demographic variations (Marchildon, 2013; Smith and Papanicolas, 2012). Lessons from these systems, including strategies to address rural–urban divides and gender disparities, could further enhance the equity and efficiency of Sweden’s healthcare delivery, particular in relation to its neighbouring countries Finland and Denmark (Satokangas et al., 2023b).

The reliance on registry data introduces potential biases, including under-reporting or miscoding of procedures. Additionally, the study does not account for patient-reported outcomes or satisfaction, which are critical for assessing the true values of surgical interventions. Future research should integrate clinical outcomes and patient perspectives to provide a more holistic understanding of trends in joint replacement surgery.

Moreover, while this study identifies significant disparities, it does not explore the underlying drivers, such as socioeconomic status, comorbidities or healthcare policy differences. Investigating these factors could offer insights for reducing inequities. Finally, as the predictive modelling suggests declining rates, future studies should monitor these trends to validate the projections and explore the factors influencing the observed declines.

## Conclusion

This study reveals a decline in the use of joint replacements in the hand and wrist from 2008 to 2020 and hereafter a moderate increase. Women are operated more frequently than men. There are important regional variations in the incidence per 100,000 inhabitants. Future studies should monitor these trends to explore the factors influencing the observed increase in incidence lately.

## Supplemental Material

sj-pdf-1-jhs-10.1177_17531934251331360 - Supplemental material for Changing patterns in joint replacement surgery in the hand in Sweden: a population-based study of 5382 patients Supplemental material, sj-pdf-1-jhs-10.1177_17531934251331360 for Changing patterns in joint replacement surgery in the hand in Sweden: a population-based study of 5382 patients by Viktor Schmidt, Elsa Pihl, Cecilia Mellstrand Navarro and Michael Axenhus in Journal of Hand Surgery (European Volume)

sj-pdf-2-jhs-10.1177_17531934251331360 - Supplemental material for Changing patterns in joint replacement surgery in the hand in Sweden: a population-based study of 5382 patients Supplemental material, sj-pdf-2-jhs-10.1177_17531934251331360 for Changing patterns in joint replacement surgery in the hand in Sweden: a population-based study of 5382 patients by Viktor Schmidt, Elsa Pihl, Cecilia Mellstrand Navarro and Michael Axenhus in Journal of Hand Surgery (European Volume)

sj-pdf-3-jhs-10.1177_17531934251331360 - Supplemental material for Changing patterns in joint replacement surgery in the hand in Sweden: a population-based study of 5382 patients Supplemental material, sj-pdf-3-jhs-10.1177_17531934251331360 for Changing patterns in joint replacement surgery in the hand in Sweden: a population-based study of 5382 patients by Viktor Schmidt, Elsa Pihl, Cecilia Mellstrand Navarro and Michael Axenhus in Journal of Hand Surgery (European Volume)

## Data Availability

The datasets can be obtained from the NPR directly (https://www.socialstyrelsen.se/en/statistics-and-data/statistics/statistical-databases/).
