# Supplementary figures and images for: Changing patterns in joint replacement surgery in the hand in Sweden: a population-based study of 5382 patients
Source: J Hand Surg Eur Vol. 2025 Apr 12;50(9):1209–14. doi: 10.1177/17531934251331360 (PMC12446691; doi:10.1177/17531934251331360)

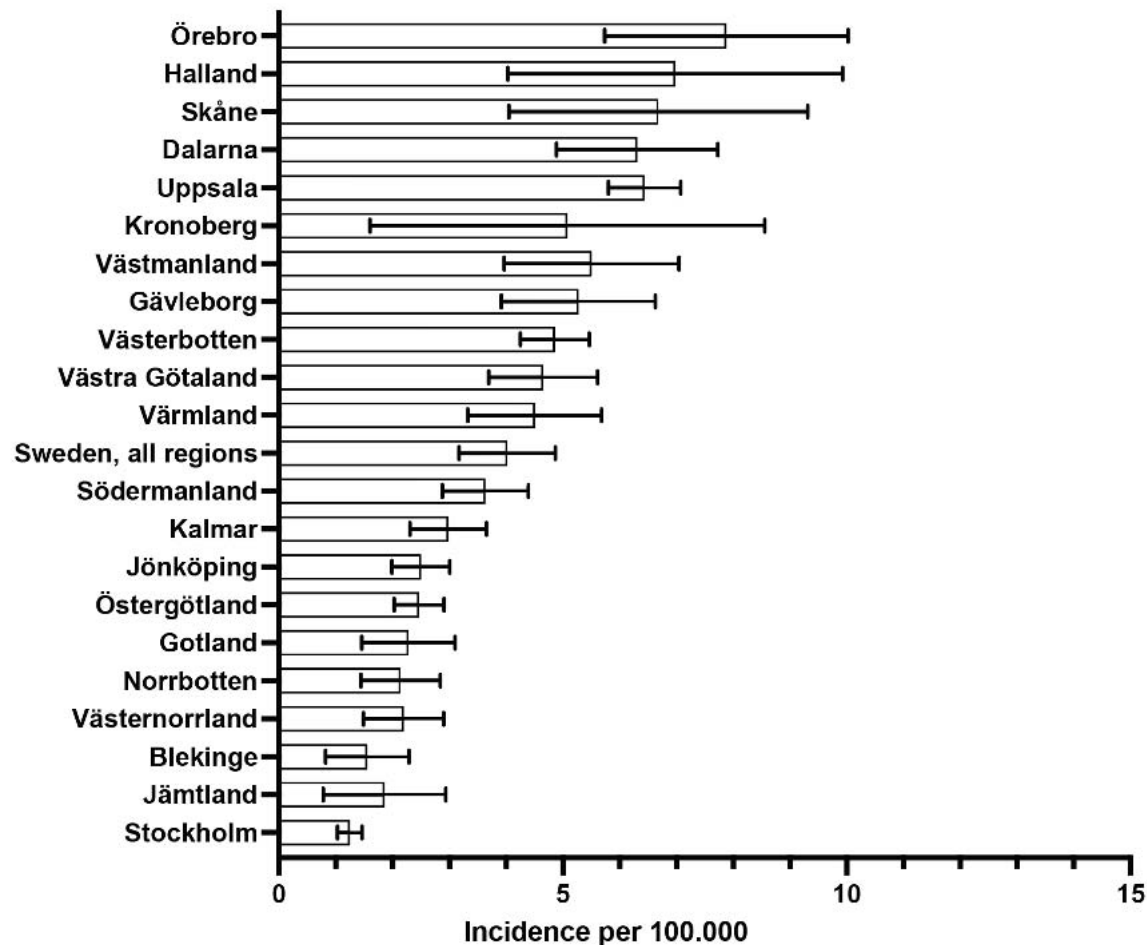

Supplement: sj-pdf-1-jhs-10.1177_17531934251331360 - Supplemental material for Changing patterns in joint replacement surgery in the hand in Sweden: a population-based study of 5382 patients [file sj-pdf-1-jhs-10.1177_17531934251331360.pdf]

A

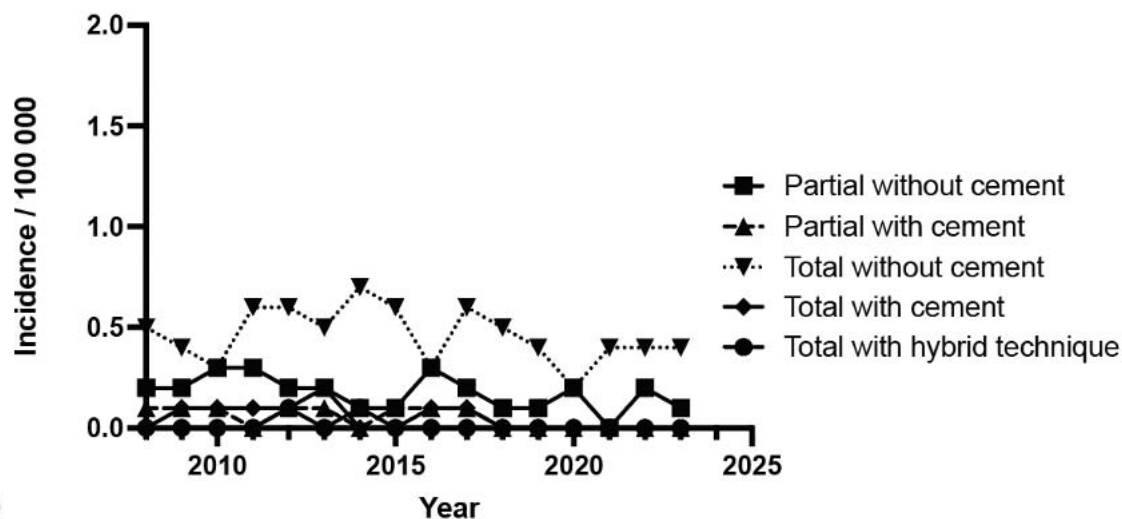

B

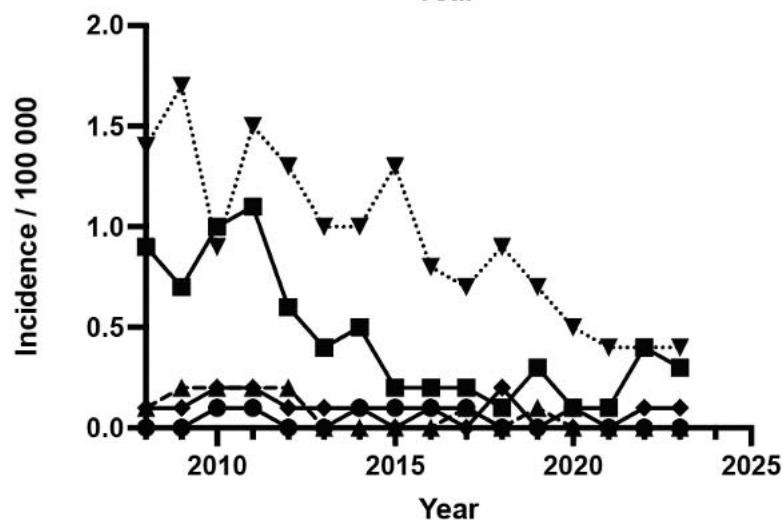

Supplement: sj-pdf-2-jhs-10.1177_17531934251331360 - Supplemental material for Changing patterns in joint replacement surgery in the hand in Sweden: a population-based study of 5382 patients [file sj-pdf-2-jhs-10.1177_17531934251331360.pdf]
