# Supplementary material for: Changing patterns in joint replacement surgery in the hand in Sweden: a population-based study of 5382 patients
Source: J Hand Surg Eur Vol. 2025 Apr 12;50(9):1209–14. doi: 10.1177/17531934251331360 (PMC12446691; doi:10.1177/17531934251331360)
Supplement: sj-pdf-3-jhs-10.1177_17531934251331360 - Supplemental material for Changing patterns in joint replacement surgery in the hand in Sweden: a population-based study of 5382 patients [file sj-pdf-3-jhs-10.1177_17531934251331360.pdf]

Supplementary *material*

Supplementary table 1: Distribution of primary joint replacement in the hand per sex and year during 2008 to 2023. Numbers are displayed as incidence per 100,000 inhabitants.

**Surgery type**	**Sex**	**2008**	**2009**	**2010**	**2011**	**2012**	**2013**	**2014**	**2015**	**2016**	**2017**	**2018**	**2019**	**2020**	**2021**	**2022**	**2023**
Primary joint replacement surgery in hand and finger joints	Men	2,7	2,8	2,9	2,4	2,5	2,6	2,4	2,1	2,3	2,2	1,6	1,4	1,2	1,6	1,6	1,8
Primary joint replacement surgery in hand and finger joints	Women	9,9	10,8	10,7	9,1	7,3	6,4	6,5	6,2	5,0	4,7	5,0	4,2	2,6	3,1	3,5	4,1
Primary partial joint replacement surgery in hand and finger joints without cement	Men	0,2	0,2	0,3	0,3	0,2	0,2	0,1	0,1	0,3	0,2	0,1	0,1	0,2	0,0	0,2	0,1
Primary partial joint replacement surgery in hand and finger joints without cement	Women	0,9	0,7	1,0	1,1	0,6	0,4	0,5	0,2	0,2	0,2	0,1	0,3	0,1	0,1	0,4	0,3
Primary partial joint replacement surgery in hand and finger joints with cement	Men	0,1	0,1	0,1	0,0	0,1	0,1	0,0	0,1	0,1	0,1	0,0	0,0	0,0	0,0	0,0	0,0
Primary partial joint replacement surgery in hand and finger joints with cement	Women	0,1	0,2	0,2	0,2	0,2	0,0	0,0	0,0	0,0	0,1	0,0	0,1	0,0	0,0	0,0	0,0
Primary joint replacement surgery in the wrist without cement	Men	0,5	0,4	0,3	0,6	0,6	0,5	0,7	0,6	0,3	0,6	0,5	0,4	0,2	0,4	0,4	0,4
Primary joint replacement surgery in the wrist without cement	Women	1,4	1,7	0,9	1,5	1,3	1,0	1,0	1,3	0,8	0,7	0,9	0,7	0,5	0,4	0,4	0,4
Primary joint replacement surgery in the wrist with hybrid technique	Men	0,0	0,0	0,0	0,0	0,1	0,0	0,1	0,0	0,0	0,0	0,0	0,0	0,0	0,0	0,0	0,0
Primary joint replacement surgery in the wrist with hybrid technique	Women	0,0	0,0	0,1	0,1	0,0	0,0	0,1	0,1	0,1	0,1	0,0	0,0	0,1	0,0	0,0	0,0
Primary joint replacement surgery in the wrist with cement	Men	0,0	0,1	0,1	0,1	0,1	0,2	0,0	0,0	0,1	0,1	0,0	0,0	0,0	0,0	0,0	0,0
Primary joint replacement surgery in the wrist with cement	Women	0,1	0,1	0,2	0,2	0,1	0,1	0,1	0,0	0,1	0,0	0,2	0,0	0,0	0,0	0,1	0,1
Primary prosthesis in carpometacarpal or metacarpal joint	Men	1,4	1,7	1,5	1,1	1,1	1,2	1,1	0,8	1,0	0,8	0,6	0,5	0,5	0,8	0,7	0,8
Primary prosthesis in carpometacarpal or metacarpal joint	Women	5,7	6,1	5,5	4,2	3,4	3,0	2,8	3,0	2,2	2,1	2,3	2,1	1,2	1,5	1,6	2,1
Primary joint replacement surgery in finger joints	Men	0,3	0,2	0,4	0,3	0,4	0,4	0,4	0,3	0,4	0,4	0,2	0,3	0,2	0,2	0,3	0,3
Primary joint replacement surgery in finger joints	Women	1,6	1,7	2,5	2,0	1,7	1,7	1,9	1,5	1,6	1,5	1,4	1,0	0,5	1,0	0,9	1,1
Other primary joint replacement surgery in finger or hand joints	Men	0,1	0,2	0,2	0,1	0,1	0,1	0,0	0,0	0,0	0,0	0,0	0,0	0,0	0,1	0,0	0,0
Other primary joint replacement surgery in finger or hand joints	Women	0,2	0,5	0,4	0,2	0,2	0,1	0,1	0,0	0,1	0,1	0,1	0,0	0,0	0,1	0,0	0,0
